# Neural Stem and Progenitor Cells in Nervous System Function and Therapy

**DOI:** 10.1155/2016/1890568

**Published:** 2016-08-14

**Authors:** Tara Walker, Jeffrey Huang, Kaylene Young

**Affiliations:** ^1^Technische Universität Dresden, 01307 Dresden, Germany; ^2^German Center for Neurodegenerative Diseases (DZNE), 01307 Dresden, Germany; ^3^Department of Biology, Georgetown University, Washington, DC 20057, USA; ^4^Menzies Institute for Medical Research, University of Tasmania, Hobart, TAS 7000, Australia

For over two decades a significant proportion of neuroscience research has been dedicated to understanding the normal function of neural stem and progenitor cells, as well as developing novel ways to use them to achieve nervous system repair. These therapeutic stem and progenitor cell-based strategies can be broadly divided into three categories: activating endogenous neural stem/progenitor cells, cell transplantation, and the use of stem or progenitor cells to model disease. The progress being made in each of these key areas is addressed briefly within this review and by key contributions to this special issue.

Within the adult mammalian brain, including the human brain, neural stem cells (NSCs) are found in the subventricular zone and hippocampal dentate gyrus, where they divide and give rise to new neurons, in a process termed adult neurogenesis. These newly generated neurons are highly plastic and are important for many brain functions including learning and memory and mood. NSCs in the subventricular zone are also capable of generating astrocytes and oligodendrocytes [1, 2], and oligodendrocyte progenitor cells, which reside in all regions of the central nervous system, continue to generate new myelinating oligodendrocytes throughout life [3]. The activity of these proliferating populations markedly decreases with ageing [3, 4] and correlates with the age-related decline in cognitive performance. However, the fact that a large pool of quiescent precursor cells can be activated in the neurogenic niches of aged mice [5, 6], as well as the fact that oligodendrocyte progenitor cells are capable of spontaneously regenerating oligodendrocytes to replace myelin lost due to central nervous system injury or demyelination [7], offers hope that the endogenous pool of neural stem and progenitor cells can be activated to generate new cells even in the aged or injured nervous system. In this special issue, L. Harris et al. (2016) extensively review the biology and potential therapeutic applications of NSCs in the developing and adult cerebral cortex.

Studies in rodents have shown that behavioural interventions such as environmental enrichment and cognitive training and exercise can promote neurogenesis [8], and some types of learning have been shown to increase oligodendrogenesis [9]. Furthermore, a number of hormones, cytokines/chemokines, and growth factors have been shown to influence endogenous cell generation, including vascular endothelial growth factor, brain-derived neurotrophic factor, nerve growth factor, progesterone, and epidermal growth factor. In many cases these identified protein regulators have poor clinical efficacy due to poor stability, an inability to cross the blood-brain barrier, or significant off-target effects on other cell types; however it is possible that the next generation of drug-design and drug-delivery approaches will overcome some of these hurdles. In this special issue, L. Auderset et al. (2016) highlight the influence that the members of the low density lipoprotein receptor related protein family and their ligands have on neural stem and progenitor cell behaviour, and A. E. Cole et al. (2016) highlight the potential of bone morphogenic protein 4 and small molecule substitutes to direct endogenous neural stem and progenitor cells to generate glial cells following a central nervous system insult. The close proximity of the NSCs to the brain microvasculature also allows them to interact with peripheral immune system, a research area highlighted by O. Leiter et al. (2016).

The second major avenue of cell-based neural repair research is stem cell transplantation, which has been used for other clinical purposes since the 1960s. Stem cells from a variety of sources have been proposed and tested for the treatment of neurodegenerative disease. While mesenchymal stem cells have a limited ability to generate neural cell types, human embryonic stem cells can be expanded* in vitro *and retain their ability to differentiate into each of the major neural cell types. However, the benefits observed in response to stem cell transplantation in mouse models of neurodegenerative disease are often not the result of the transplanted cells differentiating into functional neurons or glia on a large scale. Instead they appear to promote neural regeneration by the secretion of paracrine factors. Neural and mesenchymal stem cells that are transplanted into mouse models of Alzheimer's dementia produce beneficial neurotrophins, which have an anti-inflammatory effect and reduce both amyloid and tau pathologies. Similarly, NSCs transplanted into rodents with experimental autoimmune encephalomyelitis, a model of inflammatory-mediated demyelinating disease, have been shown to attenuate inflammation and promote functional recovery [10]. The past decade has seen the evolution of protocols that produce more consistent and defined cell populations for transplants, making it more feasible that the cells can be engineered to maximize their paracrine influence and better abrogate disease pathology. In this special issue, X. Gao et al. (2016) have shown that NSCs engineered to overexpress glial cell line-derived neurotrophic factor enhance the immunomodulatory and neuroprotective potential of NSCs transplanted into the central nervous system of rats with chronic experimental autoimmune encephalomyelitis.

The final way in which neural stem and progenitor cells are being utilized to treat nervous system disorders is through the development of cell culture-based models of disease. For example, neural stem and progenitor cells can be differentiated into neurons, astrocytes, and oligodendrocytes in order to study specific human genetic mutations associated with neurodevelopmental or neurodegenerative disorders or expanded for the large-scale screening of novel pharmacotherapies. Human neural progenitor cells are ideally suited for these studies but are difficult to obtain. However, in this special issue, H. Fukusumi et al. (2016) demonstrate that human induced pluripotent stem cells can be derived from multiple sources, including dermal fibroblasts, cord blood cells, and peripheral blood mononuclear cells and readily differentiated into human neural progenitor cells. Moreover, N. Gunewardene et al. (2016) report that human induced pluripotent stem cells can be differentiated into neurons that are able to innervate cochlear hair cells, allowing them to be used to model auditory neuron replacement therapies* in vitro*.

This special issue highlights the rapid progress being made in neural stem and progenitor cell biology.
